# Performance evaluation of cellulose triacetate and cellulose diacetate hybrid membranes with carbon nanotube (CNT) for sustainable slaughterhouse wastewater treatment via forward osmosis

**DOI:** 10.1038/s41598-026-45066-3

**Published:** 2026-04-10

**Authors:** Hanan Moustafa Abdallah Moustafa, Muanda Mukunga Meschack, Marwa Saied Shalaby, Rehab H. Mahmoud, Hassan Ahmed Abdel Moneim Farag

**Affiliations:** 1 Biotechnology Department, Institute of Graduate Studies and Research, Alexandria University, 163 El-Horeya avenue (El-Shatby), Alexandria, 21526 Egypt; 2 Chemical Engineering Department, Faculty of Engineering, Alexandria University, Lotfy El-Sied st. off Gamal Abd El-Naser, 21928 Alexandria, Egypt; 3 Chemical Engineering Department, Engineering Research & Renewable Energy Institute, National Research Centre (NRC), Elbuhouth Street 12622, Dokki, Cairo Egypt; 4 Water Pollution Research Department, National Research Centre (NRC), Elbuhouth Street 12622, Dokki, Cairo, Egypt

**Keywords:** Cellulose triacetate, Cellulose acetate, Modified carbon nanotubes, Forward osmosis, Slaughterhouse wastewater, Nutrients recovery, Microalgae biomass production, Biotechnology, Engineering, Environmental sciences

## Abstract

Slaughterhouse wastewater (SW) contains high organic matter and nutrients, requiring sustainable treatment methods like forward osmosis (FO). This study evaluates the performance of four membranes: M1 (cellulose triacetate), M2 (M1 with carbon nanotubes), M3 (cellulose triacetate/diacetate), and M4 (M3 with carbon nanotubes) for treating SW. It reports the first-time use of CNTs in a hybrid membrane (CTA/CDA) for FO applications. Characterization showed that CNTs improved the mechanical and structural properties of M1, increasing the contact angle from 68 to 75 °C and roughness from 499.59 to 542.57 nm. However, for M3, the addition of CNTs in M4 decreased the contact angle from 88 to 77° and roughness from 773.088 to 620.001 nm. While CNTs enhanced hydrophilicity, they reduced permeability and fouling resistance due to fewer water transport channels. FTIR analysis revealed distinct stretching patterns correlating with variations in contact angles and membrane performance. The evaluation of membranes in forward osmosis (FO) comprised four phases. In Phase 1, membrane M3 excelled with 91.6% water removal and 0.32 LMH flux using 0.5 M MgCl₂, outperforming M4 at 80.84% and 0.28 LMH due to Mg²⁺ ion accumulation in M4. Phase 2 confirmed M3’s superiority with MgCl₂ among the four 0.5 M draw solutions. In Phase 3, M3 demonstrated an enhancement of 93.76% and 0.33 LMH with a 1 M solution., while M4’s performance reached 90.91% with 1 M NH₄HCO₃. Overall, low water flux was attributed to the lower circulation rates of feed and draw solutions. Phase 4 showed that M3’s water flux supported the growth of *Dunaliella salina*, while M4’s lower-salinity flux hindered it. This study explores the potential of hybrid membranes reinforced with carbon nanotubes (CNTs) for forward osmosis in treating slaughterhouse wastewater. It reveals a gap in data regarding CTA and CDA blends with CNTs, marking this as a new research area. The findings indicate that CNTs do not enhance the performance of hybrid membranes for this application; therefore, cost-effective membrane (M3) using recyclable solutes like NH₄HCO₃ present a promising solution for sustainable wastewater treatment.

## Introduction

Wastewater production from slaughterhouses and meat processing industries poses a huge problem for the environment and humans. This wastewater contains high concentrations of organic matter, nutrients, biological contaminants, and suspended solids. This requires reliable solutions, but treating this wastewater is one of the most difficult and complex tasks. In addition, the growing production of slaughterhouses and meat processing facilities due to the increase in the world’s population and intensified urbanization poses an additional challenge. It is therefore essential to find effective solutions for treating these nutrient-rich effluents from slaughterhouses in order to protect the environment while complying with regulatory standards^1,2^.

The treatment of nutrient-rich wastewater requires methods or techniques that enable water to be reused on the one hand and valuable nutrients to be recovered on the other. Conventional methods (biological treatment or coagulation–flocculation + filtration) generally fail shortly to provide effective treatment, especially since wastewater has complex compositions, as they are designed to destroy pollutants rather than recover valuable nutrients or resources from wastewater^3^.

Using FO technique (membrane process), it is possible to separate water and dissolved pollutants through a semi-permeable membrane^4–6^. This technique is used in several processes such as seawater desalination, allowing water to be obtained that can be reused. In addition, Salamanca, et al^7^. and Wang and Liu^8^ showed that FO is an important alternative in wastewater treatment, particularly due to its advantages such as low energy consumption (compared to conventional methods) and high efficiency in contaminant removal.

One of the primary objectives of sustainability improvements is the recovery of nutrients from SW. When compared to the traditional method, ammonia and other nutrients can be recovered from SW in an energy- and environmentally-friendly manner. There are several methods for recovering and removing nutrients from wastewater, but they are costly and not environmentally friendly. Studies by Saad, et al^9^. demonstrated the successful use of a bioartificial hydrogel based on sodium alginate and polyvinyl alcohol as a solid drive agent in forward osmosis. In addition, other studies have shown that the use of a “green” hydrogel made from sodium alginate and linseed gum, crosslinked with epichlorohydrin and reinforced by a semi-interpenetrating polyethylene glycol network, led to a significant improvement in both swelling capacity (5300%) and mechanical strength^10^. Furthermore, the integration of Microbial desalination cells has been used in the recovery of resources (nitrogen, phosphorus, volatile fatty acids) from wastewater^11^. In addition, the quality of struvite can be improved using novel electrochemical system, with or without nanostructured coating based on agricultural waste; and significantly reduce the concentration of heavy metals (reduction of 98% compared to direct precipitation) and selective recovery of nutrients such as ammonia^12–14^.

There are several types of commercial membranes in use, among which CA and CTA-based membranes are the most widely used, due to their good hydrophilicity, cost-effectiveness, and mechanical strength. Furthermore, they have good chlorine tolerance and biocompatibility. However, CA and CTA-based membranes may face certain limitations, such as relatively low water permeability, vulnerability to biological fouling, and low rejection efficiency for certain pollutants^15^. As a result of scientific innovation, new membranes have been developed to overcome the limitations of existing membranes, using materials such as modified carbon nanotubes (CNTs) or mixed matrix composites. According to Li, et al^16^. and Wu, et al^17^., this innovation has allowed to increase the efficiency of water recovery and the removal of specific pollutants. Among the advantages of CNTs are their intrinsic antimicrobial activity and stability, as well as their high flexibility combined with a high specific surface area^18,19^. Ihsanullah^20^and Ahmed, et al^21^. demonstrated the importance of nanotechnology, one of the best solutions for sustainable wastewater treatment, in strengthening membranes, highlighting a reduction in fouling, an improvement in mechanical strength and hydrophilicity, and an increase in membrane lifespan. By strengthening the properties and structural integrity of the membrane, CNTs facilitate the FO process by promoting efficient mass transfer. Moreover, the addition or incorporation of CNTs improves membrane properties by facilitating water permeability and reducing fouling tendencies^22–24^. This also allows the reinforcement of hybrid membranes (CA and CTA hybrid membranes for example) which represent a cutting-edge approach in terms of wastewater treatment. Nitodas, et al^25^. and Arora and Attri^26^stated that due to their anti-biofouling properties, CNTs minimize the accumulation of organic and biological materials on membrane surfaces, thus prolonging the performance and duration of membranes. In addition to the membrane, the draw solution significantly influences the forward osmosis process. Its role is evident in influencing water flux, environmental sustainability, and system efficiency. According to Warsinger, et al^27^., One of the most widely used draw solutions is magnesium chloride (MgCl₂), due to its good compatibility with FO processes and its osmotic properties. Furthermore, it is important in enhancing water flux, with the possibility of its regeneration after the process. The membrane-draw solution combination is a key factor in determining the overall efficiency of FO systems. Other draw solutes, such as ammonium bicarbonate (NH_4_HCO_3_) and sodium acetate (CH_3_COONa), have been studied in the FO process to demonstrate their positive impact on the performance of the FO system due to their potential for easy regeneration and their high osmotic pressure^28^. Thus, several studies have shown that finding the right duality of maximizing water recovery and minimizing the amount of energy consumed is a big challenge to ensure the efficiency of FO demonstrating the low energy consumption of FO compared to reverse osmosis^29–32^. Siciliano, et al^33^. and Zhang, et al^34^. stated that the chemical reaction between certain pollutants in water (magnesium, ammonium, and phosphate ions) was the basis for the formation of struvite. These researches and approaches have helped to leverage secondary resources and align with the principles of the circular economy.

FO processes also offer the possibility of recovering struvite in order to reuse it in other sectors, such as agriculture, for example. Moreover, the right membrane-draw solute-feeding solution combination contributes not only to water reuse but also to the production of a valuable slow-release fertilizer, struvite^35^. Forward osmosis doesn’t just handle wastewater; it actually pulls out and concentrates important nutrients like nitrogen and phosphorus. That matters a lot for sustainable biotech work. Additionally, the obtained nutrient-rich solution can function well as an inexpensive and efficient substrate for microalgae growth^36^.

Due to lack of published data on blends of cellulose diacetate (CDA) and cellulose triacetate (CTA) with or without CNTs for treatment of slaughterhouse wastewater via FO, the aim of this study is to examine the effect of using several cellulose acetate-based membranes: commercial CTA (M1), CTA with CNT (M2), a hybrid membrane (CTA/CDA) (M3), and M3 reinforced with carbon nanotubes (CNTs) (M4) for treatment of slaughterhouse wastewater via forward osmosis (FO). The aim is extended to the use of the recovered water and nutrients for subsequent microalgae biomass production.

## Materials and methods

### Materials

CTA with CDA and CNT were used as the main components of the prepared membranes. CTA membrane was provided from National research Center, Egypt. CNT was purchased from Sigma Aldrich. The solvents employed for membrane casting were acetone and dioxane. Those materials were purchased from Egyptian society for membrane technology and its applications, National research center, Cairo, Egypt. Magnesium chloride hexahydrate (MgCl_2_.6H_2_O), ammonium bicarbonate (NH_4_HCO_3_), glucose (C_6_H_11_O_6_), and sodium chloride (NaCl) were purchased from Piochem (Egypt), and were used to prepare draw solutions of varying concentrations. The purity of the chemicals was ≥ 98.5%. Simulated wastewater was used as the feeding solution for the experiments. Saline green microalga *D. salina* from culture collection of Hydrobiology Lab, Water Pollution Research Department, National Research Centre (NRC), Egypt, was used in this study.

### Membranes preparation for FO

Four different membranes were prepared: M1, M2, M3, and M4. The composition of each membrane is shown in Table 1.

**Table 1:** FO membrane composition.

Membrane type	Composition				
	CTA (wt%)	CDA (wt%)	CNT (wt%)	Acetone (wt%)	Dioxane (wt%)
**M1 (commercial)**	12	0	0	35	53
**M2**	11	0	0.05	35	53.95
**M3**	11	1	0	35	53
**M4**	12	1	0.05	35	52.95

A doctor blade set to a thickness of 200 μm was then used to deposit the casting solutions onto glass substrates. After that, the phase separation was induced through the transfer of the freshly cast membrane into a distilled water bath (non-solvent). Once the coagulation process complete, fresh distilled water was used to thoroughly wash the membranes, until further characterization. Moreover, CNT was added at a fixed concentration of 0.05 wt%, based on a previous study that showed the optimum CNT concentration to be added in the FO system must be in the range from 0.01 to 1.0 wt.%^37^.

### Membrane characterization

The characterization of the fabricated membranes was carried out to evaluate their properties such as mechanical, chemical and structural. The analysis of the surface and cross-sectional morphology, as well as the determination of the pore structure, the distribution of modified carbon nanotubes (CNTs) and functional groups, were performed using scanning electron microscopy (SEM) and FTIR for chemical analysis^31,38,39^. The insight into the surface topography and the roughness of the membrane was performed using atomic force microscopy (AFM)^31,40^, while the hydrophilicity of the membranes was assessed through the water contact angle measurements^41^. Finally, a nano-tensile testing machine was used to determine the mechanical performance (tensile strength and elongation at break) of the membranes^42,43^. The aforementioned measurements were performed using different instrumental techniques. Fourier Transform Infrared Spectroscopy (FTIR) was conducted using a Bruker-Vertex70 FTIR with OPUS software from Bruker Optics. The detector employed was deuterated triglycine sulfate (DTGS), and the system included an accessory featuring Attenuated Total Reflectance (ATR) with a Golden Gate diamond. This setup operated over a range of 4000 cm^− 1^ to 600 cm^−1^with a resolution of 2 cm − 1 − 1 and an error margin of ± 0.01 cm^− 1^.

Scanning Electron Microscopy (SEM) was performed using a QUANTA FEG-250. Mechanical testing of the prepared membranes involved recording the stress at break (σ) and elongation at break (ε_br) on samples measuring 100 mm in length and 25 mm in width, using an H5KS universal tensile testing machine.

Atomic Force Microscopy (AFM) was conducted using the Wet-SPM model from Shimadzu, Japan. The water contact angle was assessed with a compact video microscope (CVM), adhering to the ASTM D724–99 standard test method for determining the surface wettability of paper, as well as ASTM D5946-96 for corona-treated polymer films.

### FO experiment and performance evaluation

**Fig. 1:**
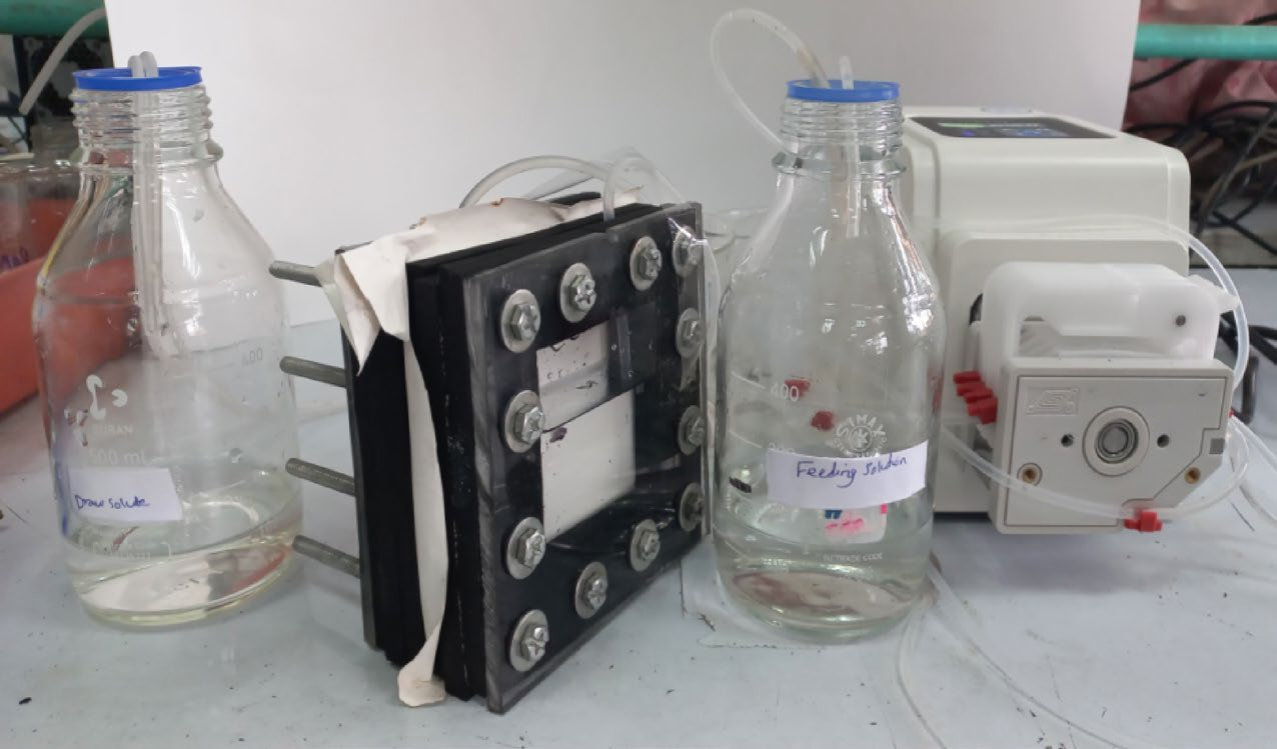
FO system for nutrients recovery.

The fabrication of the lab-scale FO system for water recovery was locally fabricated using neoprene rubber and acrylic sheets (Fig. 1). Two compartments made up the cell: a feed solution chamber and a draw solution chamber, separated by a FO membrane, where active layer of all membranes faced the feed solution. Both feed and draw solution were circulated using precision peristaltic pump at relatively low circulation rate of 18 mL/min which is the maximum flow rate obtained from this pump. A minimum of three membrane samples were used for all separation performance measurements. All FO system experiments were carried out at room temperature.

A hundred mL of both feeding and draw solutions were respectively placed on both sides of the membrane unit. The percentage of water removed (%) is a key indicator of process efficiency, it was calculated using the following Eq. (1):

1 $$\:Water\:removed\:\left(\%\right)=\frac{{W}_{d,t}-{W}_{d,0}}{{W}_{f,0}}\times\:100$$

$\:{\mathrm{W}}_{\mathrm{d},\mathrm{t}}$ and $\:{\mathrm{W}}_{\mathrm{d},0}$ are the weights of draw solution after a certain time t of filtration process, and at time zero, respectively (g). $\:{\mathrm{W}}_{\mathrm{f},0}$ is the weight of feeding solution at time zero (g).

The water flux of the FO membrane was calculated by the change in the solution volume on the liquid side. The Eq. (2) was used to calculate the water flux (Jw).

2 $$\:\mathrm{J}\mathrm{w}=\frac{{\mathrm{W}}_{\mathrm{d},\mathrm{t}}-{\mathrm{W}}_{\mathrm{d},0}}{{{\uprho\:}}_{\mathrm{w}}\times\:\mathrm{A}\times\:\mathrm{t}}$$

Jw is the water flux (LMH), $\:{{\uprho\:}}_{\mathrm{w}}$ is the water density (g/L), A is the effective surface area of the membrane (m^2^), and t is the duration of the filtration process (h).

### Application of recovered water and nutrients for microalgae biomass production

The microalgae culture was first grown in BG11 medium (containing 120 g/L NaCl) until it reached the exponential growth phase. Prior to use, the starting culture was prepared by harvesting the cells via centrifugation at 2000 rpm for 5 min and subsequently decanting the supernatant. The nutrient recovery solution (from the feed side), obtained from both membranes (M3 and M4) after the FO process, was utilized as the sole nutrient source for cultivation (directly as obtained, without any further dilution). Batch cultivations were executed in triplicate within 100 mL Erlenmeyer flasks, each containing 50 mL of the culture medium. Cultures were maintained at approximately 25 °C and continuously illuminated with 2500 lx white fluorescent light. The initial microalgal biomass concentration was set at 0.3 OD 680. Daily samples of 1 mL were collected to monitor microalgal growth. Lipid, protein, and total carbohydrates for the produced biomass were measured according to AOAC (1995).

## Results and Discussion

### Morphologies of the fabricated membranes

**Fig. 2:**
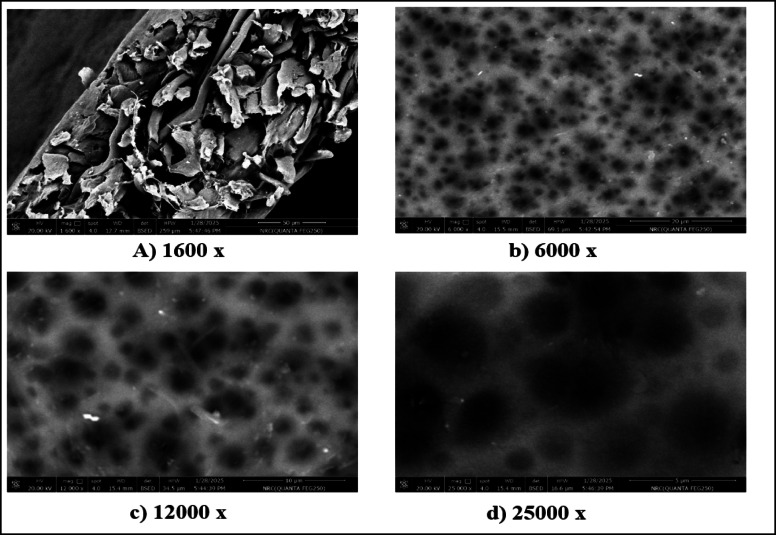
Morphology of M1 at different SEM magnifications.

**Fig. 3:**
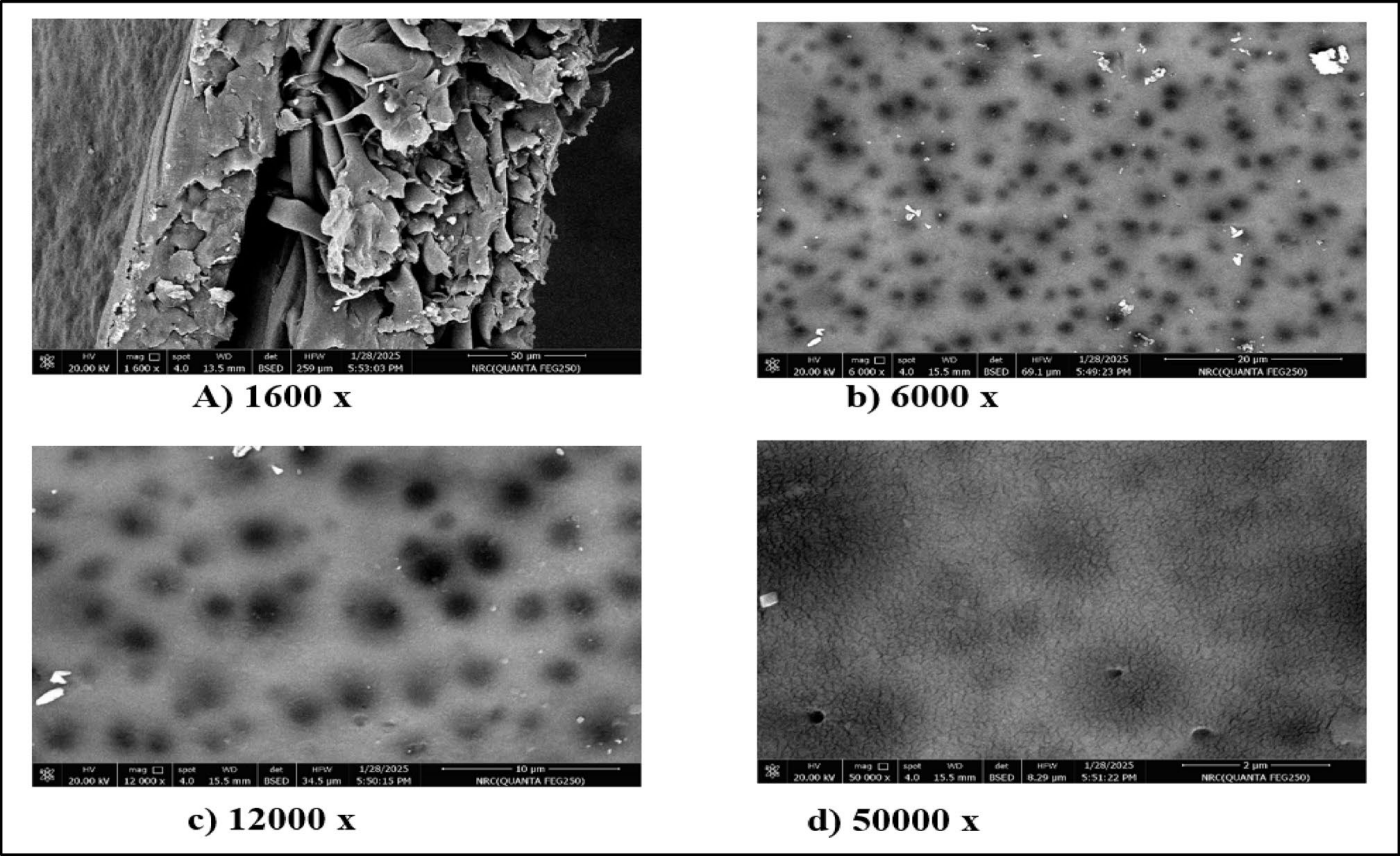
Morphology of M2 at different SEM magnifications.

Figures 2 and 3 present SEM images showing the morphologies of M1 and M2 at different magnifications. The results show that M1 exhibits a rough, non-uniform surface with irregular asperities and heterogeneous pore spatial arrangement, suggesting a less controlled polymerization process with the potential consequence of reduction of selectivity and variation in pore sizes^44–46^. The M2 membrane exhibits a denser and more uniform structure, with fewer visible defects, demonstrating that the incorporation of CNTs acts as a reinforcing agent, resulting in the promotion of tighter packing of CTA chains on the one hand and the enhancement of mechanical stability on the other hand^23,47^.

**Fig. 4:**
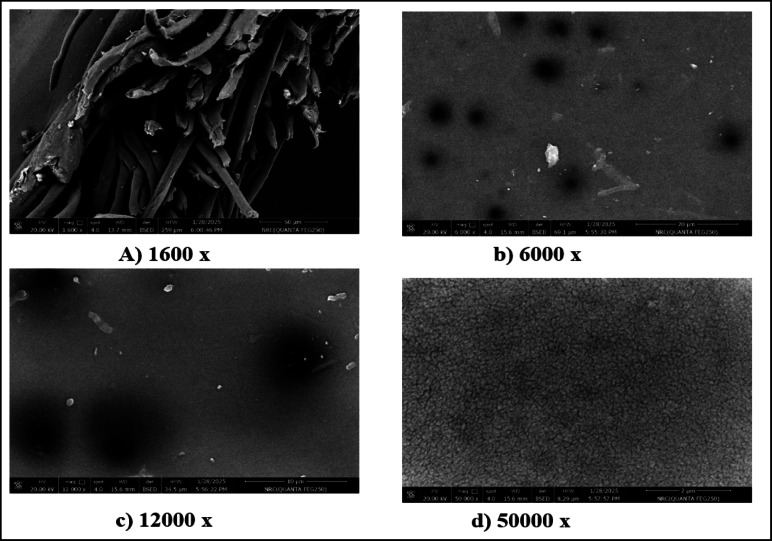
Morphology of M3 at different SEM magnifications.

**Fig. 5:**
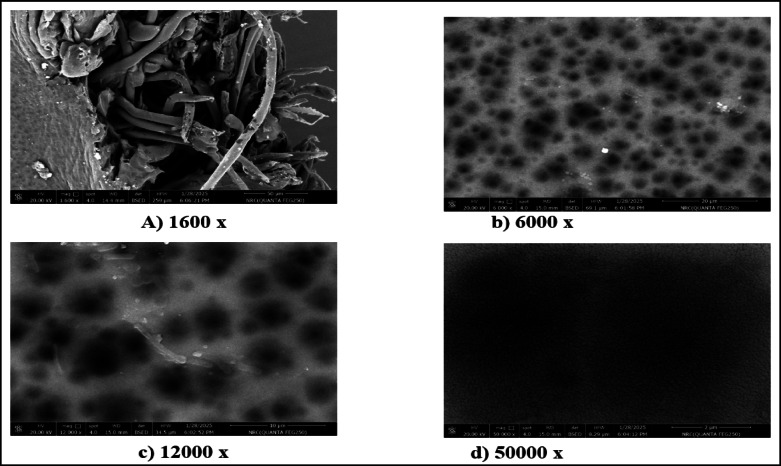
Morphology of M4 membrane at different SEM magnifications.

The small and uniformly distributed pores of M2 show that the CNT-induced nucleation during polymerization led to the refined pore morphology^48,49^. Also, in M2, the pores are small and uniformly distributed over the surface, resulting in the creation of complex molecular sieves that favor the diffusion kinetics of CO_2_ over CH_4_^50,51^. On the other hand, the incorporation of CNTs into M1 improves mechanical properties, tensile strength, and structural homogeneity. This suggests the good adhesion between polymer chains in M2 resulting in the smoother surface, while the reduction of agglomerations and voids occur in M1.

SEM images showing the morphologies of M3 and M4 membranes at different magnifications are presented in Figs. 4 and 5. According to the results, it can be seen noticed that M3 has a stratified skin and dense layer, typical of phase-inversion membranes, which confirms the findings of Xu, et al^4^. and Nguyen, et al^40^.. The microstructural characterization at 10 μm and 5 μm scales reveals small pores distributed uniformly across M3 smooth surface, showing a precisely tuned balance between the selectivity and the permeability. As founded by Kim, et al^52^., the increased surface roughness imparted by CNT promote stronger membrane-feed solution interactions. The observed structure suggests potential permeability enhancement but may negatively impact antifouling performance when CNT dispersion is suboptimal.

The integration of CNT in M3 results in an interpenetrating fibrous framework that permeates the polymeric matrix, showing the presence of an irregular pore structure featuring expanded pore dimensions alongside prominent high-contrast agglomerates and localized dark domains indicative of CNT clustering. This modification could simultaneously improve hydrophilicity, decrease crystallinity, improve structural integrity, and increase porosity while boosting mechanical stability^53–55^.

### Chemical structure of the membranes in terms of FT-IR spectra

**Fig. 6:**
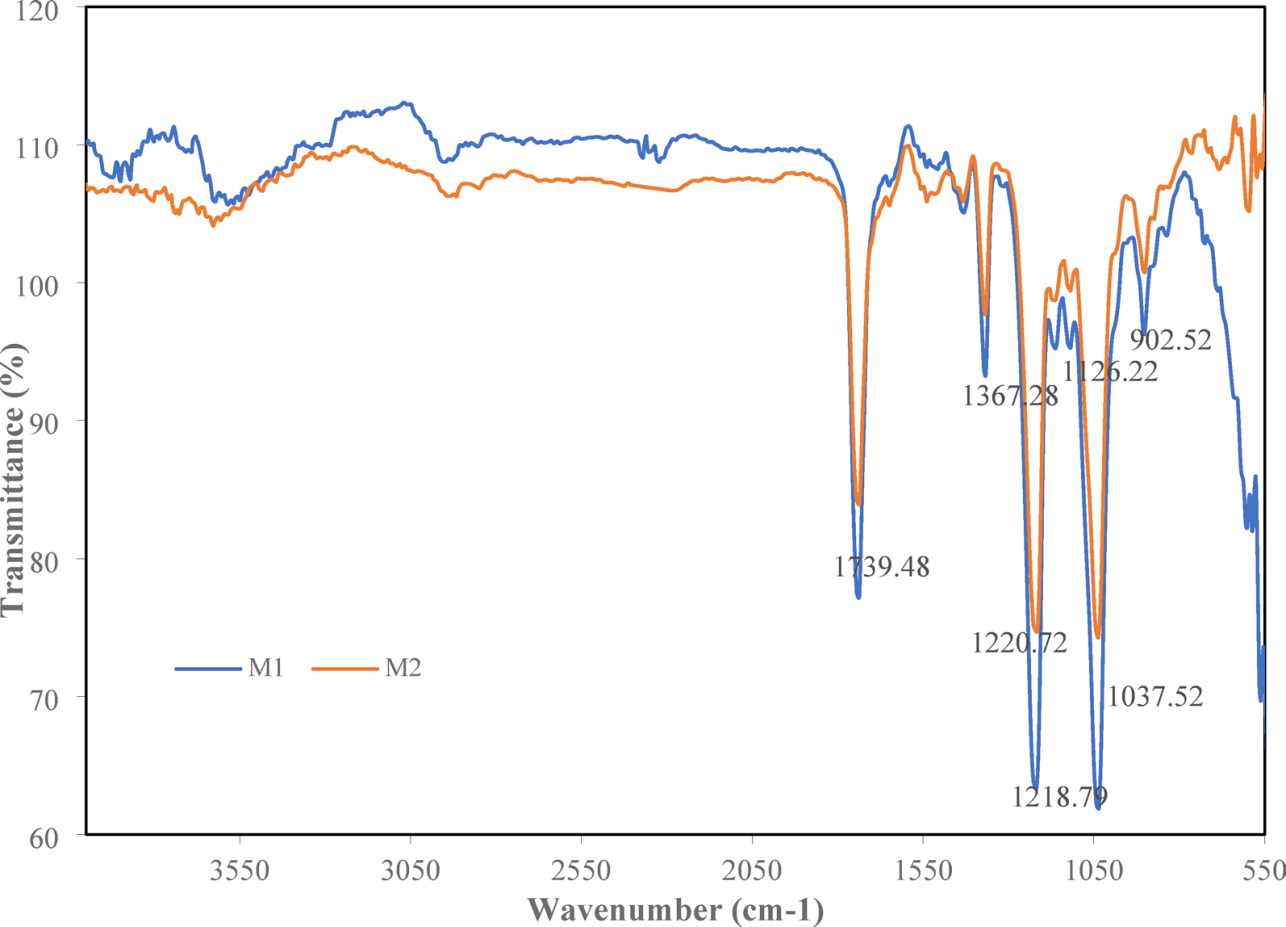
FT-IR spectra of M1 and M2 membranes.

FT-IR analysis results of the membranes (M1 and M2) are presented in Fig. 6. The results reveal C–O–C, C–O, and C = O vibrations and small amounts of free hydroxyl groups (–OH) in M1, confirming the presence of ester groups formed through the acetylation of cellulose hydroxyl functionalities on the one hand and the presence of asymmetric and symmetric stretching vibrations on the other hand, which is the intrinsic identity of M1. Also, as confirmed by Rahman, et al^56^. and Shaikh, et al^57^., the purity of M1, as well as the lack of contaminating compounds, is confirmed by the absence of aromatic or phenolic bands.

After the incorporation of CNT on M1, the FT-IR analysis shows the same peaks as M1, with differences on transmittance values.

**Fig. 7:**
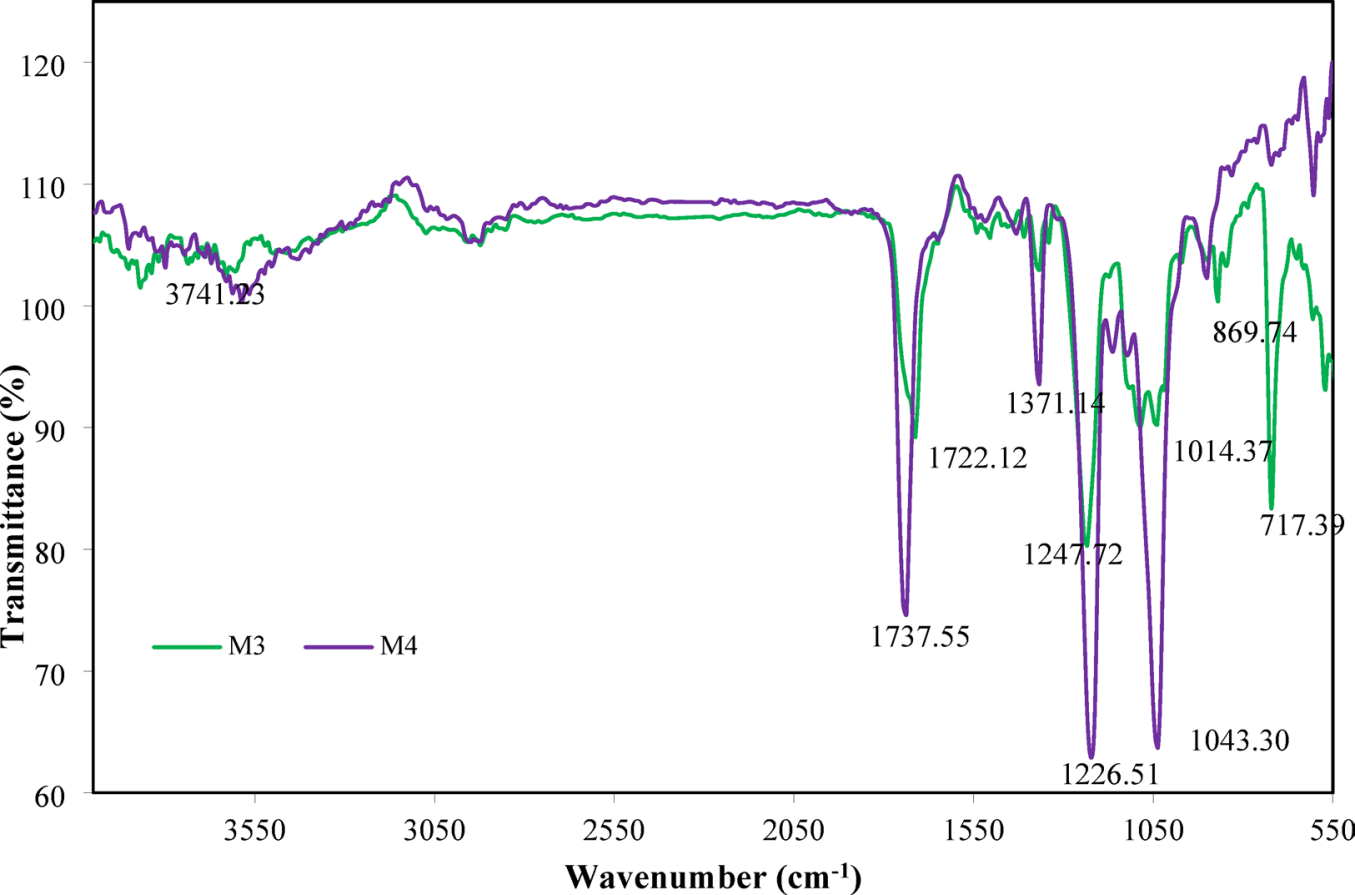
FT-IR spectra of M3 and M4 membranes.

However, a peak is observed around 1542.77 cm^[‒1^, most likely related to an amide II vibration and confirming that M1 was functionalized with a polymer containing –NH– or –CONH– groups due to the incorporation of CNT.

The results of FT-IR analysis of the membranes (M3 and M4) are presented in Fig. 7. The results show C–O–C, C–O and C = O vibrations in M3 membrane, reflecting the dominance of the CTA structure. A broad vibrational band spanning 3700–3260 cm^[‒1^ is also observed. This also confirms the acetylation of cellulose hydroxyl functionalities due to the presence of ester groups on the one hand, and the presence of unesterified hydroxyl –OH groups inherent in the CDA polymer structure on the other hand^58,59^. This demonstrates the increased hydrophilicity of the material compared to pure CTA. Due to the dual contribution of CTA and CDA in M3, there is a positive contribution to the improvement of hydrophilicity, mechanical and morphological properties, influencing the hydrogen bonding network.

After incorporation of CNT on M3, a peak is observed at 1371.14 cm^[‒1^, likely related to bending vibration of the methyl group (–CH_3_) of the acetyl moiety in CTA/CDA, potentially enhanced or slightly displaced by interaction or hydrogen bonding with functional groups of the CNT. Another peak at 603 cm^[‒1^ is observed and attributable to a metal–oxygen (M–O) bond. It is noteworthy that the non-detection of aromatic C = C around 1600–1500 cm^[‒1^, characteristic of the presence of CNTs in the matrix, is due to its low concentration (0.05%) or to spectral superposition with characteristic CTA/CDA vibrational modes. Nevertheless, the incorporation of CNTs plays a key role in modulating important intensity and position peaks, which is consistent with physical interactions such as hydrogen bonding or π-π stacking, as reported in analogous composite membranes^60–62^.

Through these FTIR results, it is obvious that the studied membranes comply with the validated principles of nanocomposite manufacturing, and sufficiently prove the strong separation potential in the FO process.

### Mechanical properties of the membranes

**Fig. 8:**
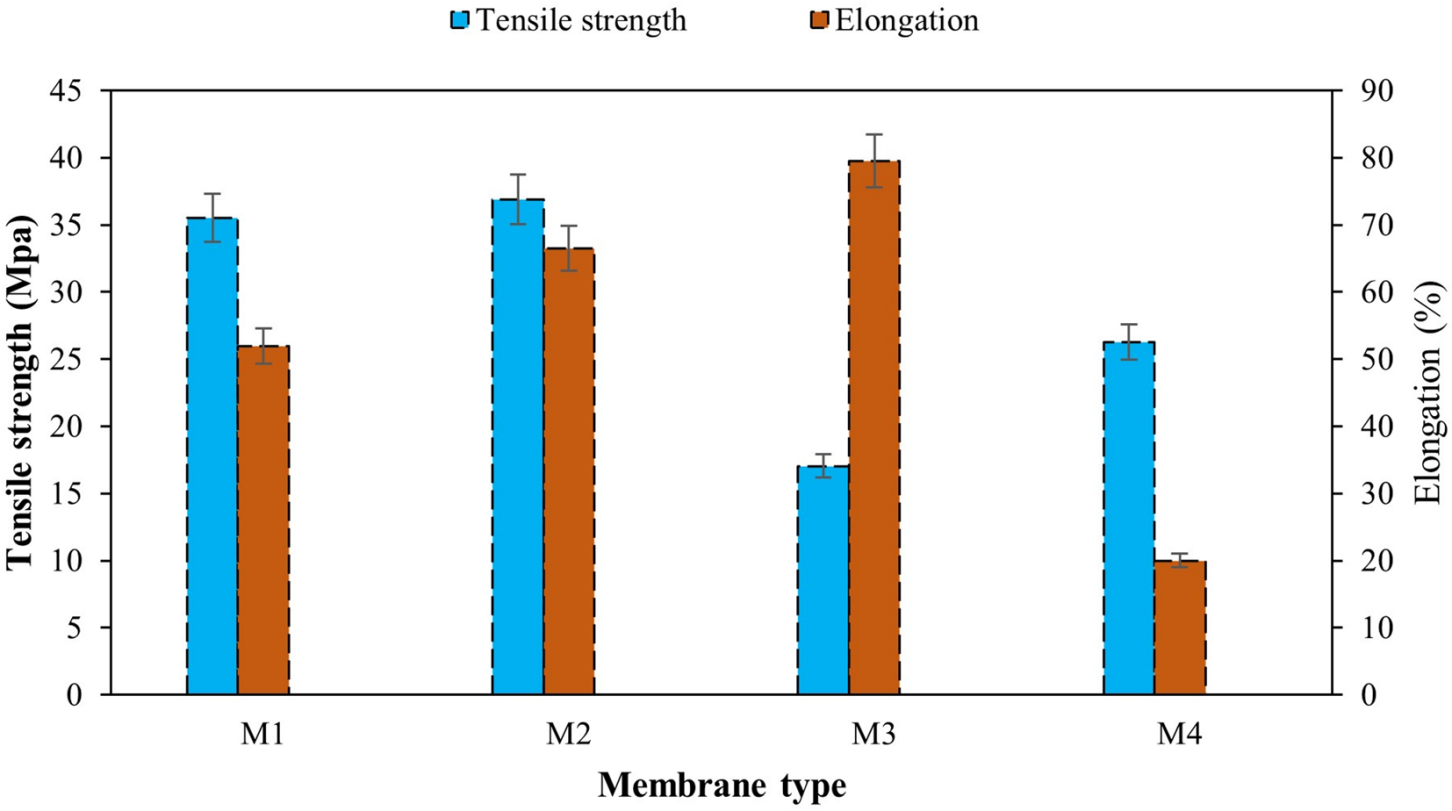
Mechanical properties of the prepared membranes.

**Fig. 9:**
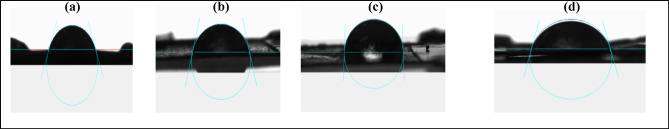
Water contact angle of M1 (a), M2 (b), M3 (c), M4 (d).

The mechanical test results of the membranes are shown in Figs. 8 and 9. The results reveal that the incorporation of CNTs enhanced the membrane’s load resistance, significantly improved its mechanical robustness and the compensation of material weaknesses introduced by CA^63^. In addition, adhesion to the polymer matrix interface was improved^64,65^.

The increase in elongation may be due to the reduction in rigidity and cohesive strength of M3 on the one hand, and the effect of the higher hydroxyl content of CA increasing flexibility on the other hand^66,67^. The CNT incorporation in CTA matrix indicates better energy dissipation and chain mobility, possibly due to weak CNT interfacial bonding. CNTs improve both strength and flexibility of the membrane.

Moreover, the addition of CNT to M3 demonstrates the restriction of chain mobility, while improving the tensile strength with limitation of elongation^68^.

### Hydrophilic properties of the membranes

**Fig. 10:**
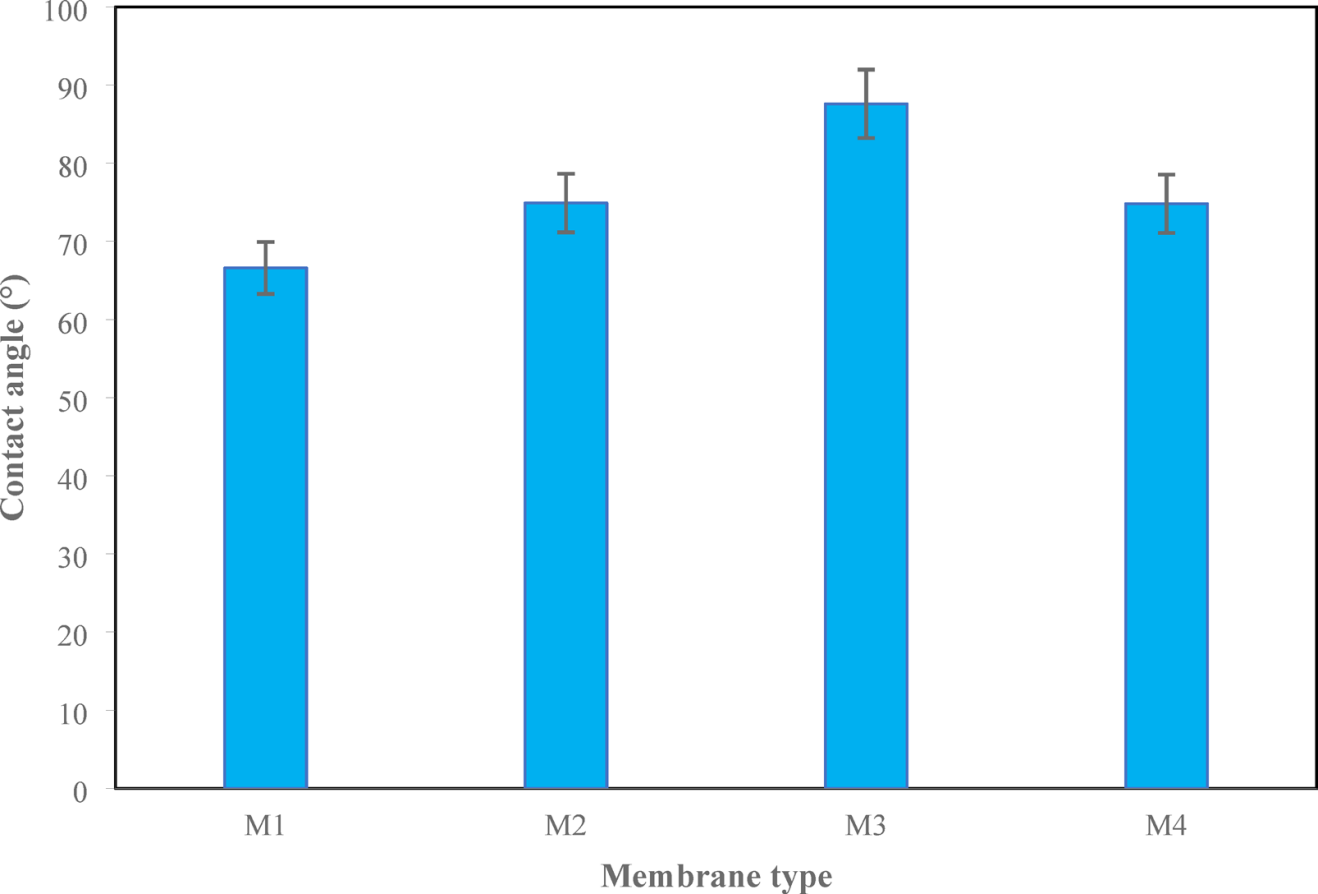
Water contact angle values of the all the membranes.

The water contact angle results of the membranes are presented in Figs. 9 and 10. The lower value of M1, confirming its hydrophilicity capacity, could be explained by the favorable wettability due to the acetylated structure and the near absence of hydroxyl groups available for hydrogen bonding^57^. The addition of CDA to CTA leads to intermolecular hydrogen bonds between the CTA and CDA chains, with three effects: first, the reduction of the number of free polar groups on the M3 surface; second, the increase of the contact angle value; and third, the limitation of the interaction of water with the surface^58,59^. The surface becomes more heterogeneous and denser, limiting the uniform dispersion of water.

Comparing M1 and M2, it is obvious that the incorporation of 0.05% CNT into the CTA matrix results in the reduced overall hydrophilicity, which may be associated with the hydrophobic character of CNT^69^. However, it can be concluded that there is good compatibility between the CTA network and CNTs, but the increase of the contact angle suggests the obscurity of partial hydrophilic surface regions by CNTs^55^. The other effects of the reduction of hydrophilic regions are the increase of mass transfer resistance, the decrease of membrane porosity and surface area^70^.

In CTA/CNT membranes, increased hydrophobicity changes to the restoration of hydrophilicity upon the addition of CDA to the CTA/CNT composite due to the relationship between surface roughness and the density of functional groups on the surface. In pure CTA, CNTs only add nanoscopic roughness at the surface, which creates air pockets that increase the contact angle through the Cassie-Baxter effect. However, when CDA is added to the CTA/CNT composite (which contains a greater number of unbound hydroxyl (-OH) functional groups), it causes hydrophobic “peaks” of the nanotubes to become scaffolds for the rapid movement of hydrophilic materials onto the surface of the nanotubes; the CDA polymer chains will either coat or stain the surface of the CNTs, thereby allowing the formation of a more rapid solvent/non-solvent exchange during the phase inversion process, improving porosity, and creating a higher polarity surface that facilitates wetting of water and decreasing the contact angle^61,71–73^.

### Hydrophilic properties of the membranes

**Fig. 11:**
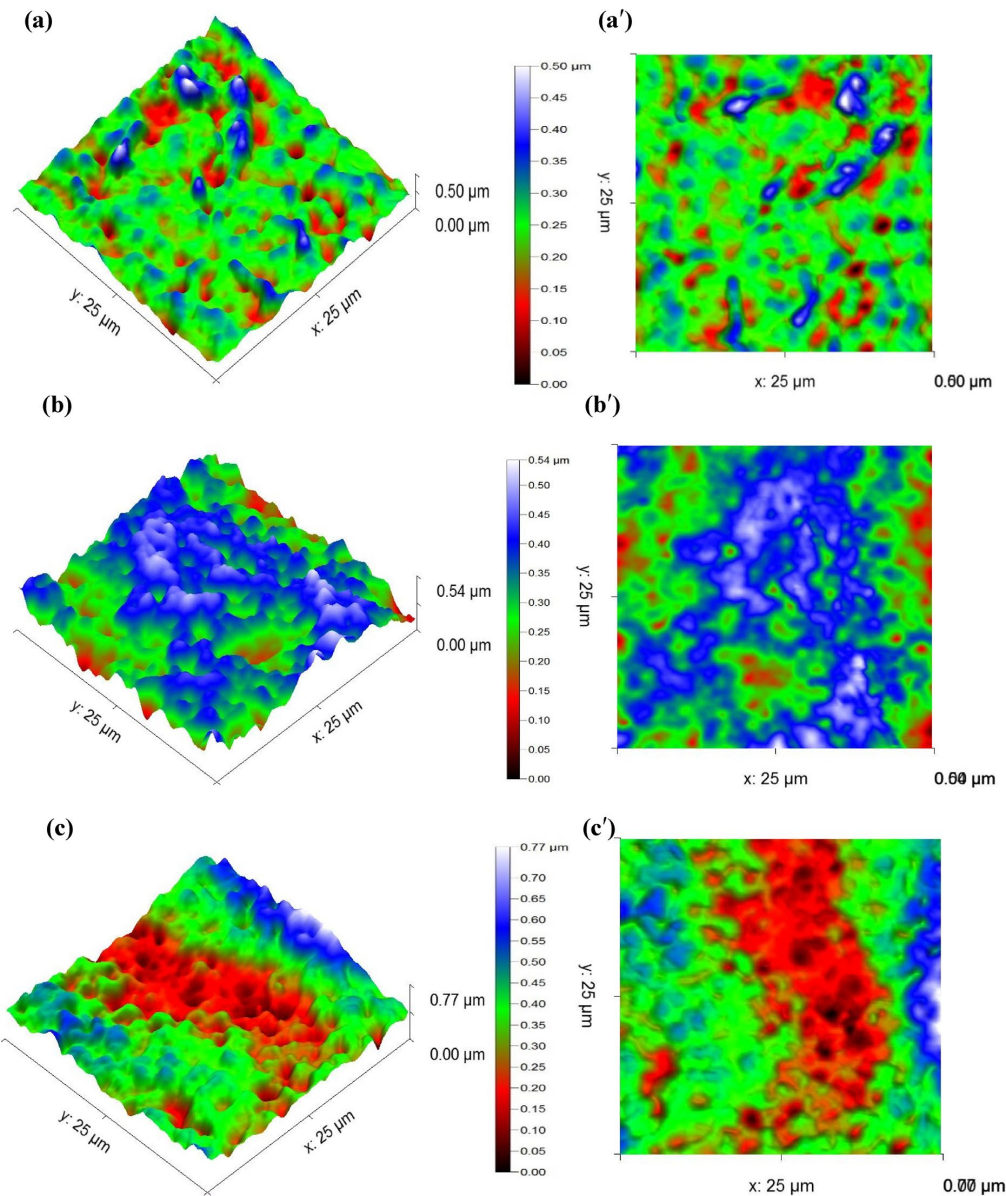

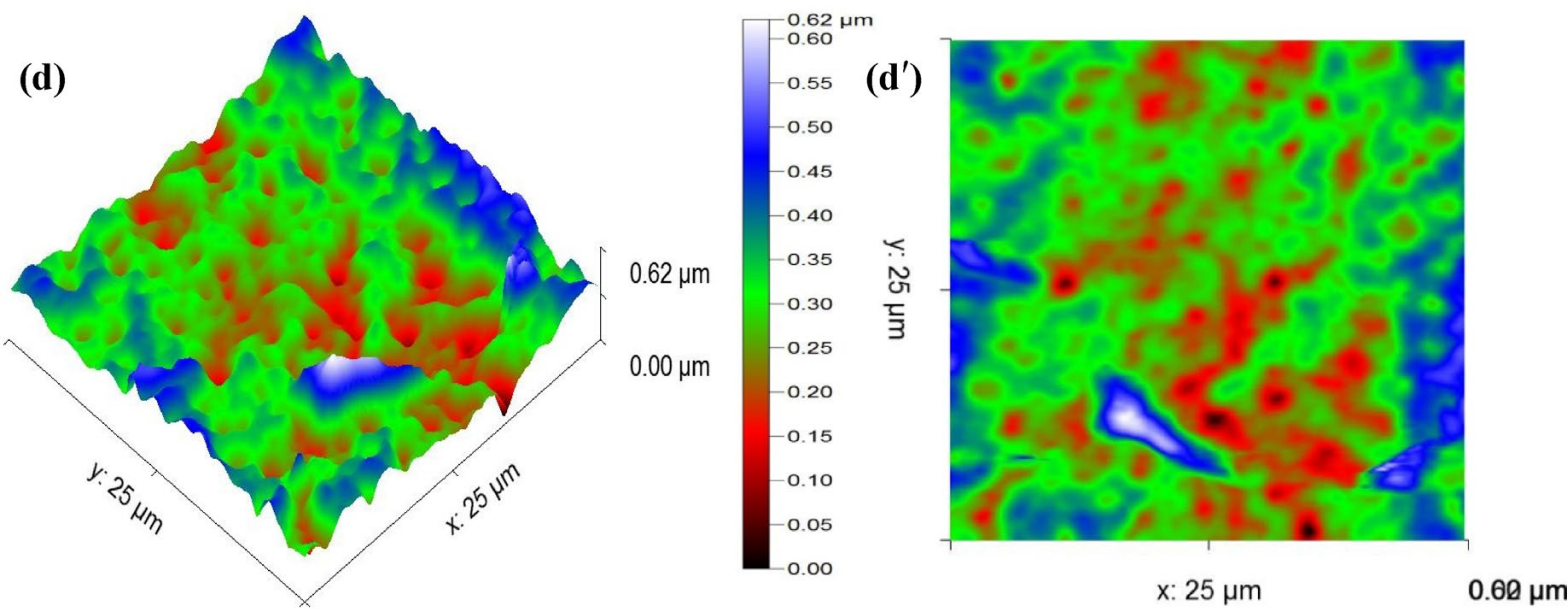
3D and 2D AFM images of the membranes (a) 3D M1, (a*′*) 2D M1, (b) 3D M2 (b*′*) 2D M2, (c) 3D M3, (c*′*) 2D M3, (d) 3D M4 (d*′*) 2D M4.

AFM images depicting the four types of membranes are presented in Fig. 11, while the corresponding surface roughness parameters are detailed in Table 2.

According to the results of Figs. 11(a) and 11(a′), the architecture of M1 exhibits a smooth surface, which shows that there is the reducing of fouling due to the moderate elevation and valleys, as well as a fewer number of anchoring sites for foulants, resulting in minimal textural complexity^74^. After the addition of CDA to CTA matrix (Figs. 11(c) and 11(c′)), it can be observed a systematic increase of the roughness of M3. This increase the irregularity of the membrane topography, resulting in the apparition of deep valleys and sharp ridges, could be explained by the inhibition of water spread by the creation of macro-texture onto the surface^58^. In addition, this also confirms the increase of the roughness due to polymer-polymer incompatibility or CTA/CDA phase separation during membrane fabrication, that increase at the same time antifouling resistance and contaminants retention^60^.

According to Figs. 11(b) and 11(b′), it is obvious that the incorporation of CNTs into CTA matrix creates micro-aggregates or protrusions. As reported by Dai, et al^75^. and Sun, et al^76^., M1 functionalized with a polymer containing –NH– or –CONH– groups after incorporation of CNT increased the roughness which has the effect of improving the antifouling performance of the membrane or reducing its clogging. After the incorporation of CNT into M3 (Figs. 11(d) and 11(d′)), a decrease in the roughness of M4 is observed. A more uniform surface topography is observed, as the CNT-bridging polymer-polymer interfaces prevent phase separation over large areas. The changes in roughness (from M3 to M4) may also suggest an improvement in the hydrophilicity of the membrane providing a good operational stability and water flux^59^.

**Table 2:** Roughness parameters of the membranes.

Membranes	Parameters		
	Sa (nm)	Sq (nm)	St (nm)
M1	42.8452	56.8853	499.598
M2	64.3455	78.7194	542.577
M3	95.96	119.475	773.083
M4	73.515	93.574	620.001

Sa: arithmetic average roughness. Sq: root mean square roughness. St: total roughness (St).

### Study the performance of the membranes

#### Water removal and water flux

**Fig. 12:**
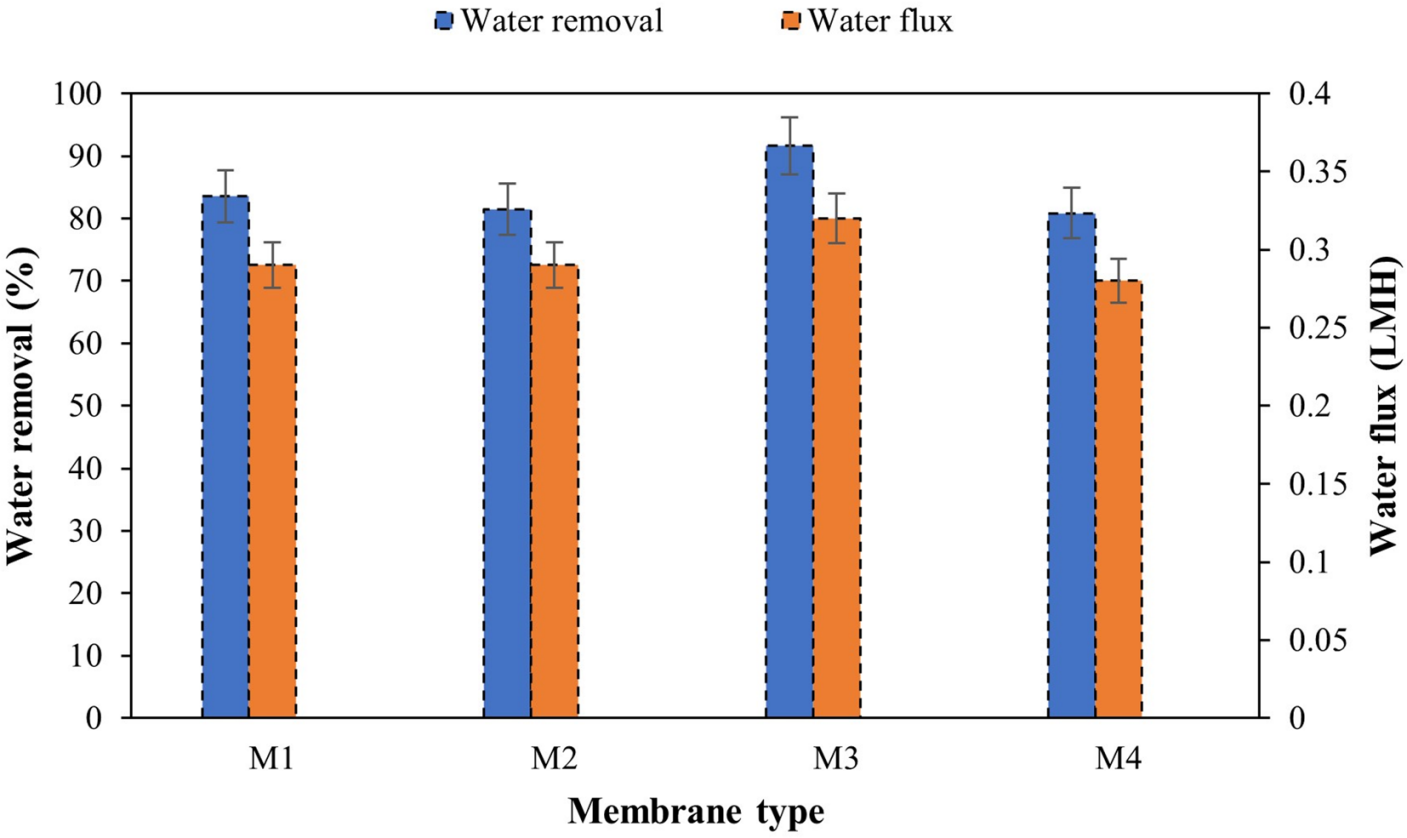
Water removal and water flux of the membranes, using 0.5 M MgCl_2_ draw solute after 24 h.

The performance of prepared membranes was evaluated by the water removal and the water flux. Figure 12 illustrates both water removal and water flux of various membrane types after 24 h using a 0.5 M MgCl₂ draw solution. According to that, the combination of CTA (11%) and CDA (1%) exhibits increased water removal and water flux, mainly due to the good chemical interaction between CTA and CDA, resulting in improved membrane porosity and hydrophilicity and increased antifouling resistance and contaminant retention. The results corroborate with the findings of^77^, confirming the improvement of M3 selectivity which enhance water transport properties and reduce mass transfer resistance.

The slight reduction in water removal due to the incorporation of CNT to M1 could be associated with the pore blockage, and as reported by Lee, et al^74^. and Seyedpour, et al^78^.. This may also cause the increase of internal concentration polarization (ICP), which limits the performance of membranes in direct osmosis (FO) and involves the accumulation of solutes from the draw solution caused by the structural changes of the membrane. The consequence is that water is less well transferred from the feed solution to the draw solution due to the reduction of osmotic water removal.

Moreover, the addition of CNT to the M3 membrane reveals a drop of both water removal and water flux. This indicates that although there is an improvement in the membrane hydrophilicity after the incorporation of CNT, a regression of membrane permeability and fouling resistance is observed, causing the creation of fewer channels for water transport and reduction of interconnected porosity, therefore less flow and less water recovery^79^.

#### Effect of draw solution type

**Fig. 13:**
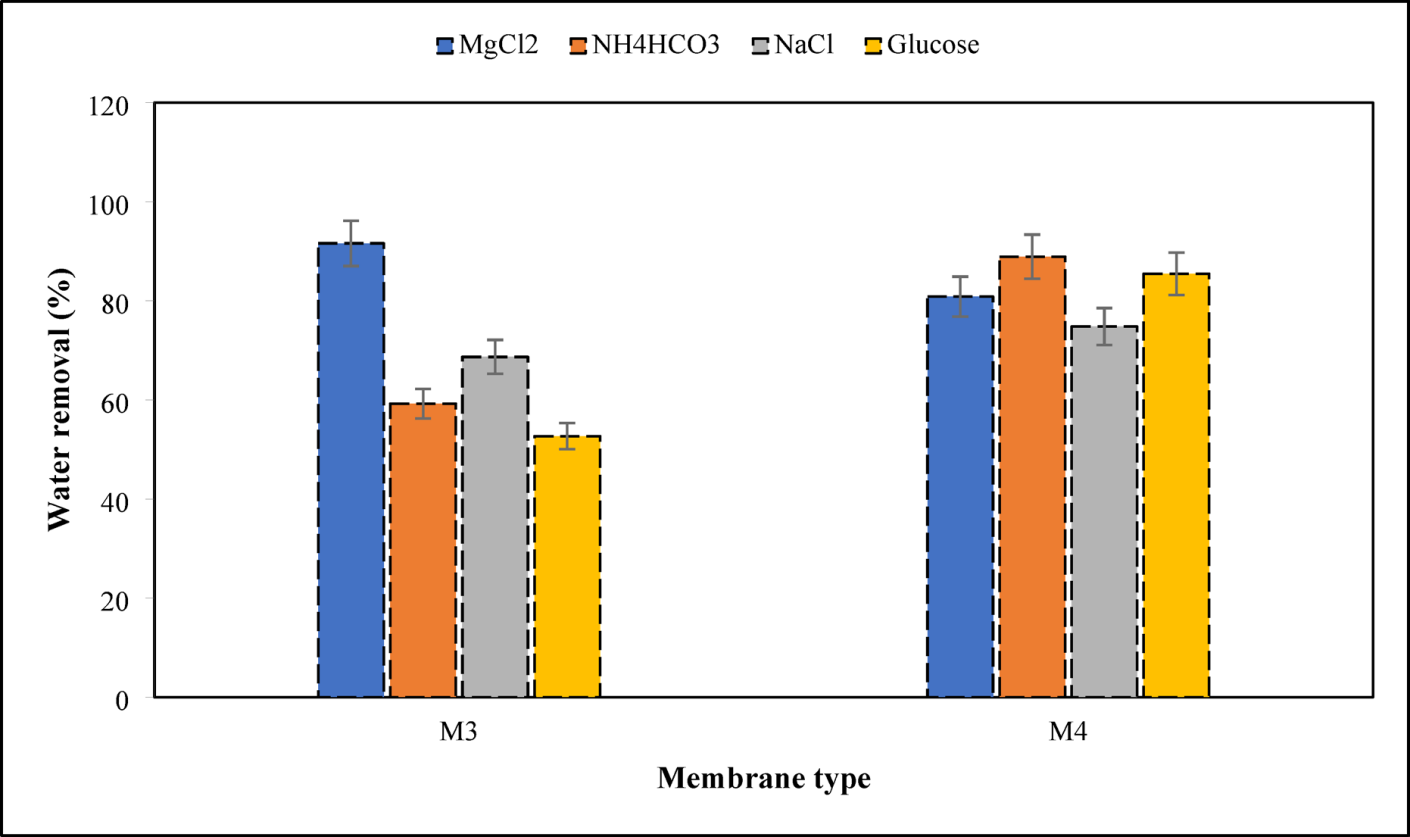
Water removal of M3 and M4, using different types of 0.5 M draw solute after 24 h.

**Fig. 14:**
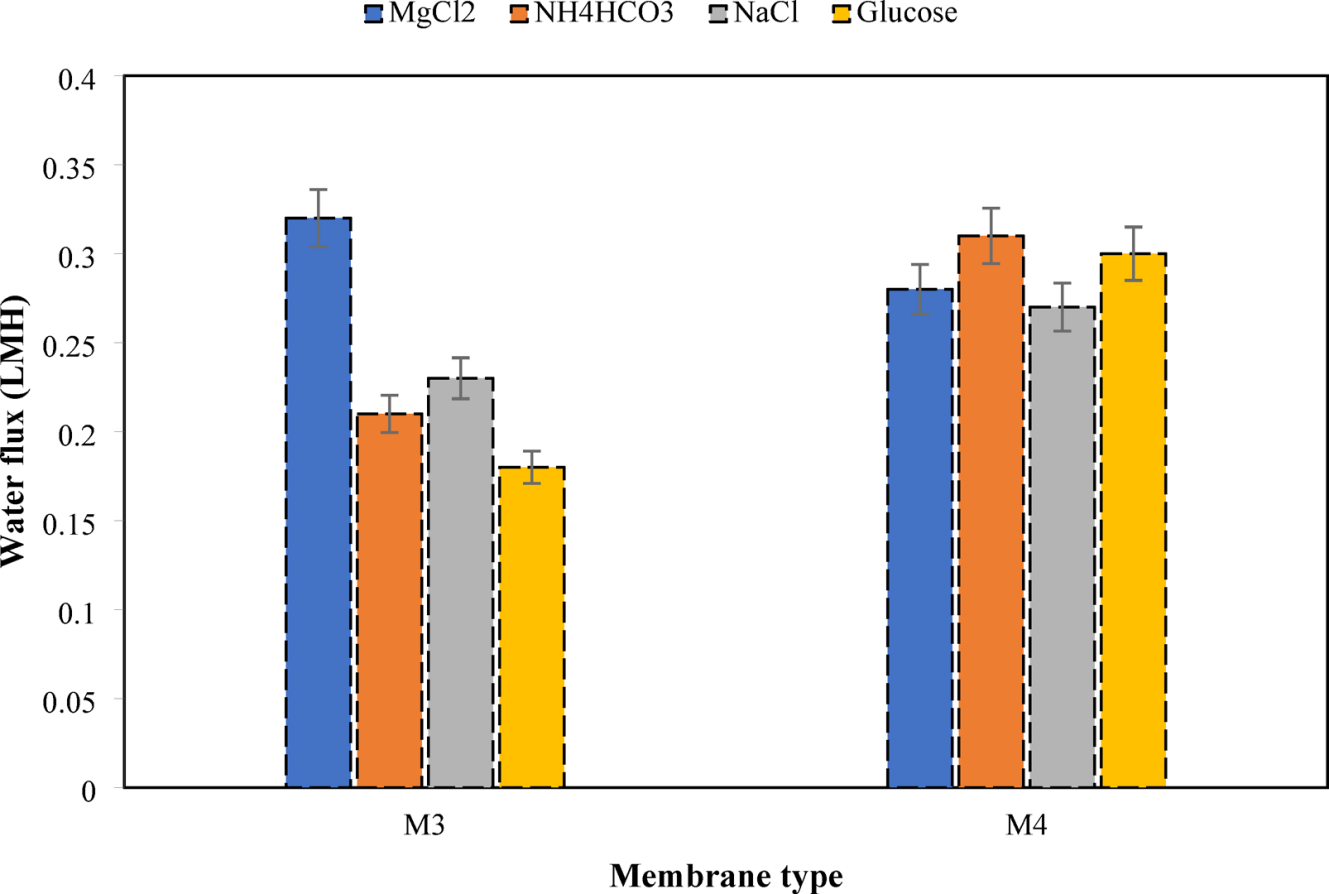
Water flux of M3 and M4, using different types of 0.5 M draw solute after 24 h.

The effects of different draw solutions (MgCl₂, NH₄HCO₃, NaCl, and glucose) are studied in this section at a concentration of 0.5 M after 24 h of operation to evaluate the performances of M3 and M4 in the FO process. The parameters assessed include water removal efficiency (Fig. 13) and water flux (Fig. 14), which together provide valuable insights into the influence of both draw solute characteristics and membrane composition on FO efficiency.

Using 0.5 M MgCl₂ as a draw solution, M3 provides the highest water removal (91.60%) and water flux (0.32 LMH) due to the good chemical compatibility between CTA and CDA, on the one hand, and to the chemical stability of the membrane in the presence of multivalent salts such as MgCl₂ without degradation or loss of selectivity, on the other hand. The stronger driving force for water permeation and osmotic pressure may be involved in the dissociation of MgCl₂ in three ions (Mg²⁺ and 2Cl⁻) according to Eq. (3)^80,81^.

3 $$\:\mathrm{M}\mathrm{g}{\mathrm{C}\mathrm{l}}_{2}\left(\mathrm{s}\right)\to\:{\mathrm{M}\mathrm{g}}^{2+}\left(\mathrm{a}\mathrm{q}\right)+2{\mathrm{C}\mathrm{l}}^{-}\left(\mathrm{a}\mathrm{q}\right)$$

The incorporation of CNT into an M3 in an FO process reveals its limited effect related to the accumulation of Mg²⁺ ions in the support layer due to their very low ionic mobility and large hydrodynamic radius, which increase the ICP^77,82–84^. Therefore, the osmotic gradient at the active layer is significantly reduced, resulting in decreased water removal and flux.

The use of 0.5 M NH₄HCO₃ as a draw solution and M₃ demonstrates that, despite the spontaneous decomposition of NH₄HCO₃ at room temperature according to Eq. (4), the reduction of the concentration of osmotically active particles in the solution occurred^85^. Due to this thermal instability, water is less attracted to the solution. The inhibition of the osmotic process was also caused by the instability of the system.

4 $$\:{\mathrm{N}\mathrm{H}}_{4}\mathrm{H}\mathrm{C}{\mathrm{O}}_{3}\left(\mathrm{s}\right)\to\:{\mathrm{N}\mathrm{H}}_{3}\left(\mathrm{g}\right)+{\mathrm{C}\mathrm{O}}_{2}\left(\mathrm{g}\right)+{\mathrm{H}}_{2}\mathrm{O}$$

Therefore, the easier diffusion of ions (Eq. (5)) due to their small hydrodynamic radius leads to an increase in the reverse flux of these ions, and thus, M3 acts as a non-selective membrane. This phenomenon reduces the osmotic pressure of the draw solution and contaminates the feed solution, resulting in a reduction in the water flow rate.

5 $$\:{\mathrm{N}\mathrm{H}}_{4}\mathrm{H}\mathrm{C}{\mathrm{O}}_{3}\left(\mathrm{s}\right)\to\:{\mathrm{N}\mathrm{H}}_{4}^{+}\left(\mathrm{a}\mathrm{q}\right)+{\mathrm{H}\mathrm{C}\mathrm{O}}_{3}^{-}\left(\mathrm{a}\mathrm{q}\right)$$

However, the incorporation of CNTs into the CTA/CDA matrix appears to have systematically improved membrane properties, increasing water removal and water flux. The low osmotic strength of NH_4_HCO_3_, compensated by CNTs, reduces water diffusion resistance and increases the hydrophilicity, softness, and permeability of the membrane. In addition, the generation of $\:{\mathrm{N}\mathrm{H}}_{3}$ and CO_2_ in the solution may lead to microbial growth, but the use of M4 increases the antifouling properties. Thus, CNTs make the membrane more selective for water removal and increase its retention of other solutes, leading to a more stable osmotic gradient and reduced ICP^74,79^.

The results for NaCl with M3 show moderate water removal and water flux. Compared to MgCl₂, which releases 3 ions (Eq. (3)), NaCl in monovalent salt form generates fewer particles (2 ions). In addition, the Na + ion is small and highly mobile, allowing it to diffuse rapidly through the membrane, reducing the action of osmotically active ions during suction and resulting in greater reverse leakage. The water removal efficiency is therefore low and unstable. The addition of CNTs results in an increase in water removal and flux. CNTs do not drastically alter the ionic barrier for smaller monovalent ions like Na⁺ and Cl⁻, unlike larger or multivalent solutes (Mg²⁺), so Na⁺ passes more easily and ion leakage persists.

The non-electrolytic state of glucose (draw solution) and its low osmotic pressure led to limited and poor performance with M3, confirming the findings of Achilli, et al^80^. which indicated that organic solutes in the FO process are highly dependent on the osmotic contribution on the one hand, and on the interaction between molecules and the membrane on the other hand. Moreover, the probable reaction of glucose with the hydrophilic groups of M3 may locally alter the membrane permeability and thus contribute to reducing the water flux over time. Also, the large dimension of the glucose molecule allows it to diffuse slowly through the membrane, thus limiting the reverse solute flux.

However, the addition of CNT significantly increases the performance of the membrane. This is explained by the fact that the presence of CNT opens more porous and interconnected channels significantly increasing the membrane’s affinity for water due to the functionalized groups (–OH, –COOH, etc.)^86^. Thus, the effective osmotic gradient is better preserved, thus increasing the water flux. Even though water removal and water flux increase significantly, the osmotic pressure remains low due to the non-electrolytic state of glucose^87,88^. The use of MgCl_2_ showed best results using M3, while NH_4_HCO_3_ showed best results using M4.

#### Effect of draw solution concentration

**Fig. 15:**
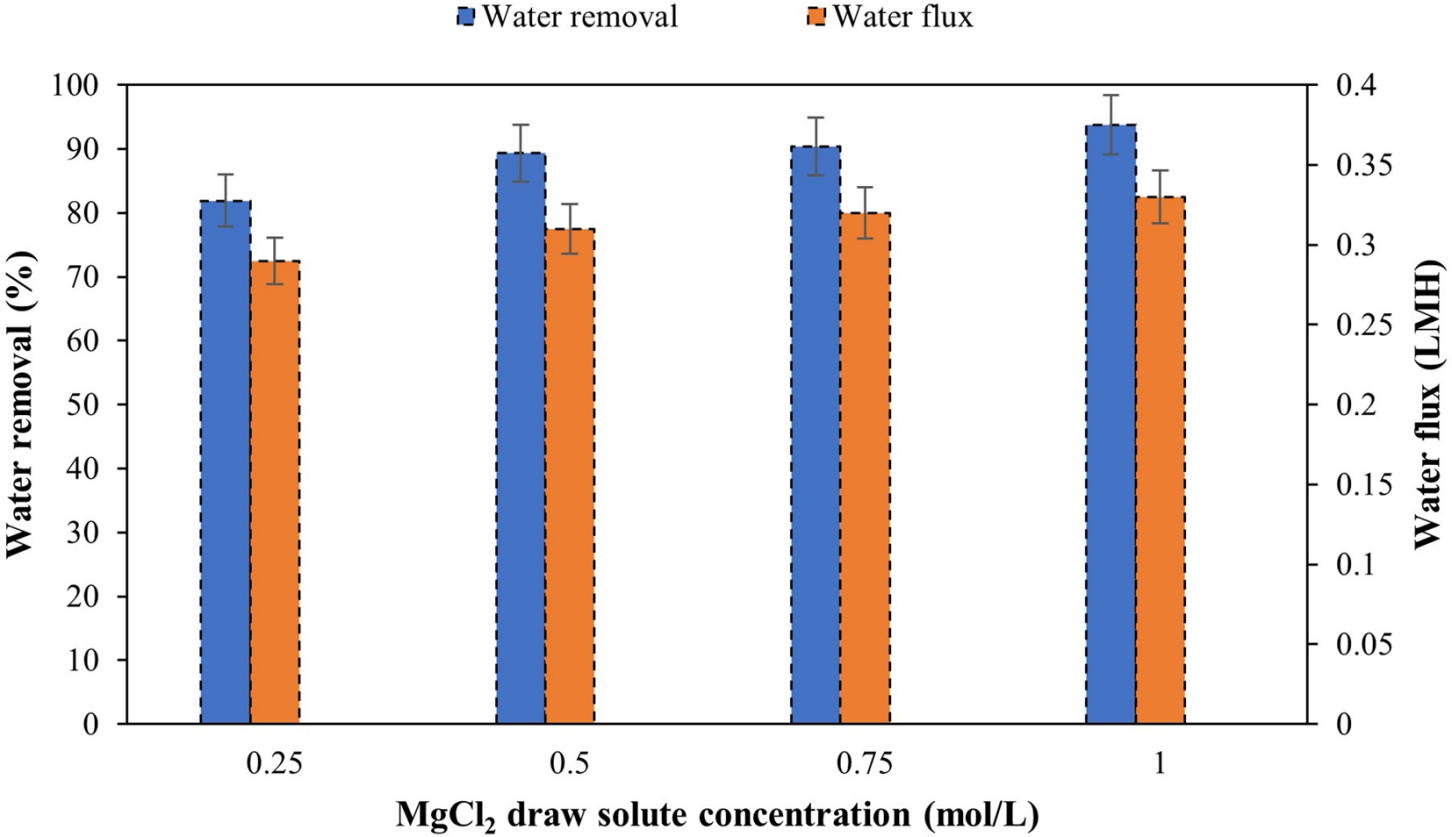
Water removal and water flux of M3 using different concentrations of MgCl_2_ draw solute after 24 h.

**Fig. 16:**
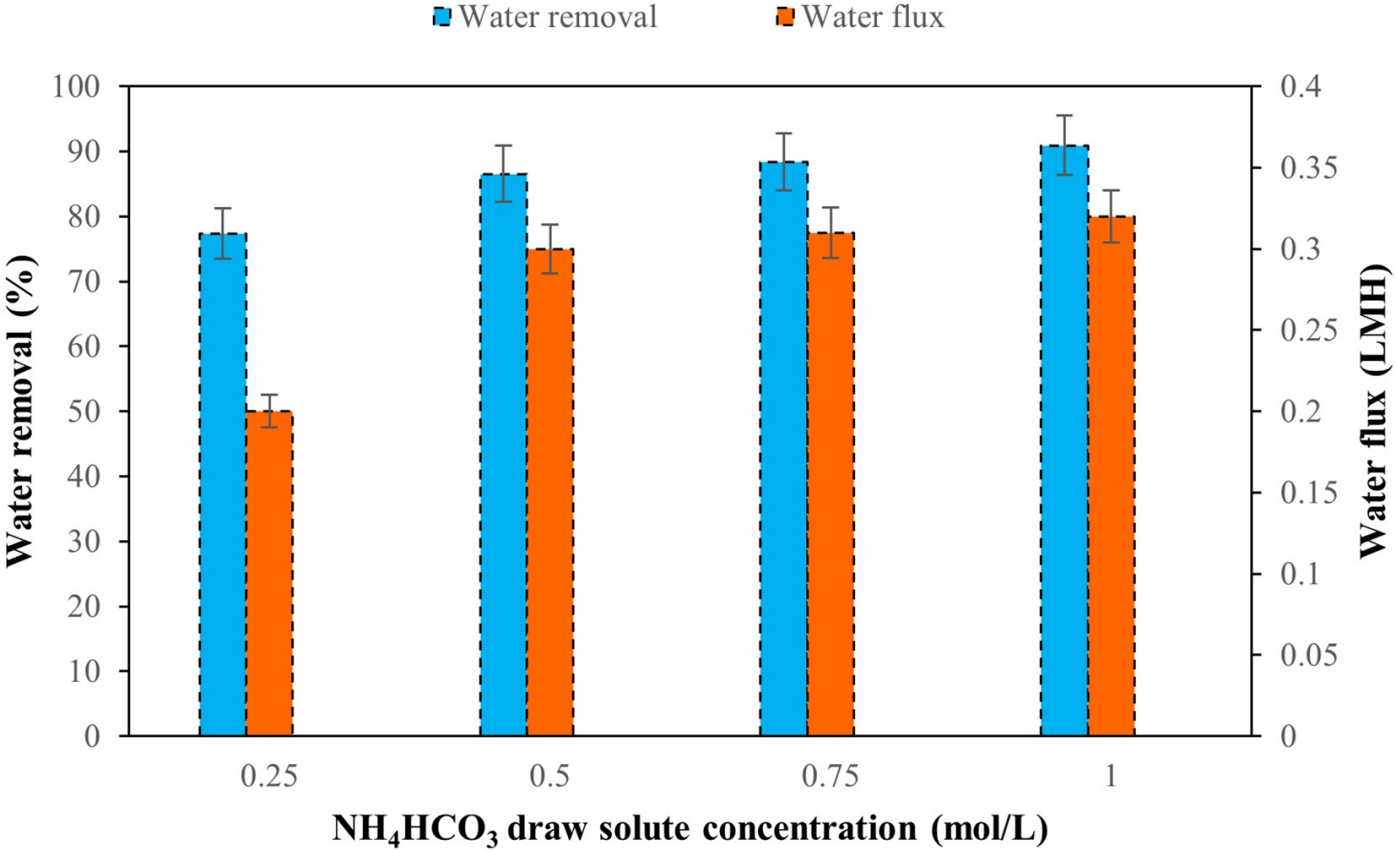
Water removal of M4 using different concentrations of NH_4_HCO_3_ draw solute after 24 h.

Figures 15 and 16 show results of MgCl_2_ and NH_4_HCO_3_ draw solutions concentrations using M3 and M4, respectively, in FO process after 24 h.

As shown in Fig. 15, water removal and flux increase with the concentration of the draw solution. This is explained by the fact that M3s are composed of hydrophilic polymers capable of forming hydrogen bonds with water, thus allowing its passage while retaining solutes (selective permeability) and increasing the osmotic pressure (π). The Van’t Hoff equation (Eq. (6)) shows the direct proportionality between the osmotic pressure and the molar concentration of the draw solution^2^.

6 $$\:{\uppi\:}=\mathrm{i}\times\:\mathrm{C}\times\:\mathrm{R}\times\:\mathrm{T}$$

where i, C, R and T are the dissociation factor (Van’t Hoff), the molar concentration of draw solution, the gas constant, and the absolute temperature (K), respectively.

MgCl_2_ is dissociated into three ions (1 Mg^2+^ + 2 Cl^‒^), i = 3. And according to Fig. 15, going from 0.25 M to 1 M, the osmotic pressure quadruples because Mg²⁺ creates a zone of high osmotic pressure at the support/active layer interface. The osmotic pressure gradient between the feeding and draw solutions is maintained for longer periods due to the low reverse leakage of Mg^2+^.

A similar phenomenon of increased performance is observed with the use of different concentrations of NH_4_HCO_3_ and M4 (Fig. 16). The incorporation of CNT improves the hydrophilicity of the membrane by creating a more open porous structure. The membrane then effectively exploits the increase in the osmotic gradient with reduced ICP^89,90^. Through this incorporation, M4 acts as a barrier against small molecules and eliminates the risk of reverse saline flux (due to the small size of the $\:{\mathrm{N}\mathrm{H}}_{4}^{+}$ ion and its high mobility). As the NH_4_HCO_3_ concentration increases from 0.25 mol/L to 1 mol/L, the effective water removal increases, from 77.32% to 90.91%. The water flux increases from 0.2 LMH to 0.32 LMH.

However, at very high NH_4_HCO_3_ concentrations, the efficiency of FO process may be affected due to the affected due to the increased viscosity of the draw solution reducing the membrane permeability by fouling^47,91^.

### Microalgae biomass Production

**Fig. 17:**
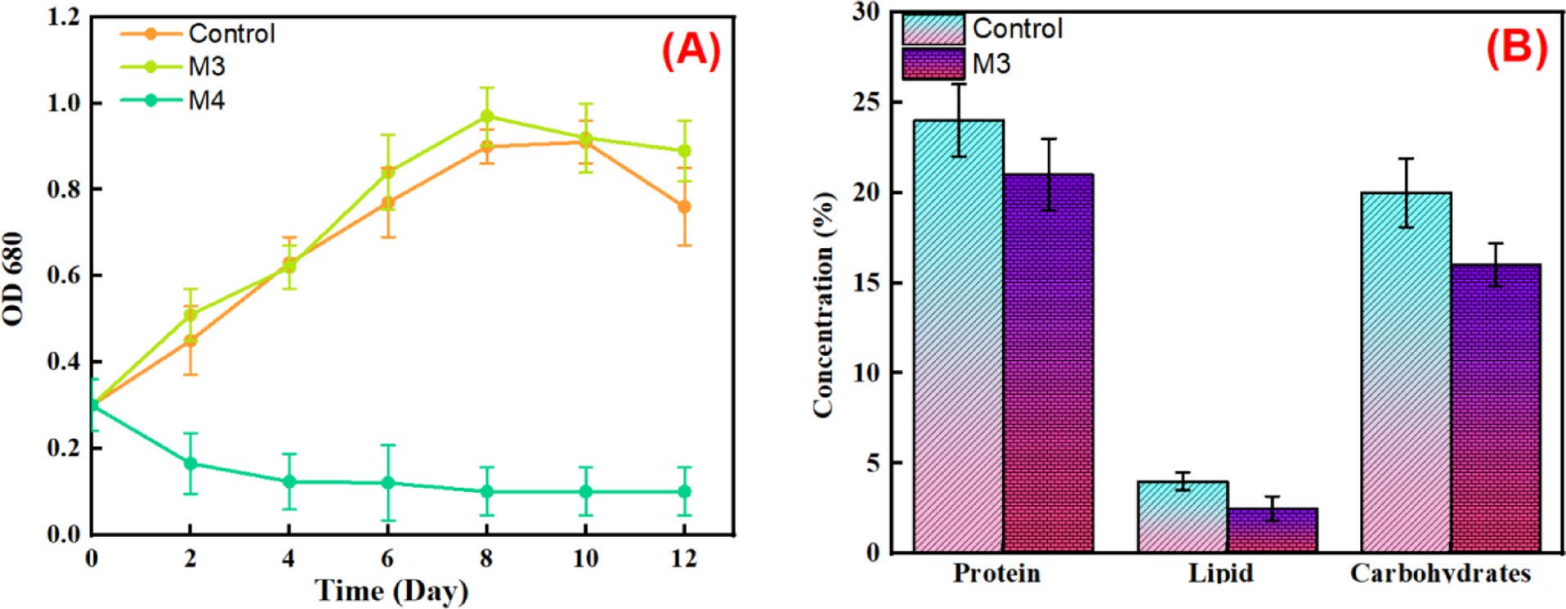
*D. salina* (A) growth curves over 12 days, monitored by OD _680_, and (B) biochemical composition (Protein, Lipid, and carbohydrate) % w/w of dry biomass cultivated using water flux recovered from M3 and M4 compared to the microalgae growth on BG11 as a control.

*Dunaliella salina* is a unicellular, marine phytoplankton classified within the phylum Chlorophyta, family Dunaliellaceae. Originally described over a century ago, the *Dunaliella* genus is recognized as a key primary producer in hypersaline environments globally^92^. The results presented in Fig. 17 provide crucial insights into the potential of membrane-recovered water flux to support microalgae cultivation with the electrical conductivity (EC) of the recovered solution emerging as the primary limiting factor.

Figure 17A illustrates the growth kinetics of D. salina over a 12-day period across three distinct cultivation conditions: a standard control medium and water flux recovered from membranes M3 and M4. Notably, *D. salina* cultivated in the water flux from membrane M3 demonstrated robust growth, closely mirroring the growth profile observed in the control medium. Both conditions exhibited a clear exponential growth phase, reaching maximum optical densities OD680 of approximately 0.9 by day 8, followed by a stationary or slight decline phase towards the end of the cultivation period. This strong performance is directly attributed to the favorable salinity of the M3-recovered solution. Specifically, the electrical conductivity (EC) of the M3 water flux was measured at approximately 17 ± 3 mS/cm. This concentration level is highly suitable for the hypersaline nature of *Dunaliella* salina, allowing it to thrive optimally.

Conversely, *D. salina* cultivated in the water flux recovered from membrane M4 exhibited a severely inhibited growth pattern, with OD_680_ values remaining consistently low (below 0.2) throughout the entire 12-day period. The underlying reason is the significantly lower EC of the M4-recovered solution, which was measured at only 4 ± 0.96 mS/cm. This low salinity likely resulted in osmotic stress for the halotolerant *D. salina*, which requires a high-salt environment for turgor pressure regulation and survival, thus preventing successful proliferation. This comparison confirms that the membrane’s ability to retain the necessary salinity (nutrients/ions) dictates the biological outcome.

Figure 17B details the biochemical composition of the *D. salina* biomass under these conditions, specifically focusing on protein, lipid, and carbohydrate content. Consistent with the growth data, *D. salina* grown in the water flux from membrane M3 showed a biochemical profile comparable to the control, with protein content around 21%, lipids about 2.5%, and carbohydrates approximately 16%. This balanced composition further supports the conclusion that the M3-recovered water flux provides a suitable environment for microalgal growth and metabolite production. In contrast, while the M4 condition resulted in minimal biomass, any measurable composition would likely be skewed or indicative of stress, if detectable at all. The lower lipid content in both M3 and control conditions compared to carbohydrates and protein is typical for microalgae grown under non-stress conditions that prioritize general growth over lipid accumulation.

## Conclusion

Slaughterhouse wastewater is a strong industrial effluent rich in organic matter (blood, lipids, oils, and grease), nitrogen, and phosphorus, needing stringent, multi-stage treatment. In this study, 4 membranes were used from M1 to M4. M1 is a commercial CTA, M2 is M1 with CNT, M3 is a CTA/CDA hybrid membrane, and M4 is M3 enforced with CNT. All membranes were characterized through SEM, FTIR, and mechanical testing. The addition of 0.05 wt% CNTs enhanced surface smoothness and selectivity. Antifouling and permeability improved, indicated by an 8.3° increase in contact angle, and hydrophilicity reduced for M2. Performance evaluations using 0.5 M MgCl₂ showed M3 achieving 91.6% water removal and 0.32 LMH flux, while M1 achieved 83.55% and 0.29 LMH. CNT incorporation slightly decreased flux due to pore clogging from structural changes. Subsequent tests indicated M3’s performance varied with different draw solutions, with MgCl₂ demonstrating superior results. Experiments revealed that increasing draw solution concentrations to 1 M enhanced water removal and flux, with M3 performing optimally with MgCl₂ (81.89–93.76% removal, 0.29–0.33 LMH flux). M4 also performed well with NH₄HCO₃ (77.32–90.91% removal, 0.2–0.32 LMH flux). Both membranes showed promise for slaughterhouse wastewater treatment, improving hydrophilicity and permeability, but M3 is better than M4. Accordingly, there is no need to introduce CNT in the M3 membrane, and the hybrid membrane (CTA/CDA) is sufficient for the treatment of slaughterhouse wastewater. Using a recyclable draw solution such as NH₄HCO₃ provided a feasible solution for closed-loop operations. The generated water flux was able to support microalgal growth, with flux from M3 showing better performance, indicating potential for integrated nutrient and water recovery.

## Data Availability

No datasets were generated or analysed during the current study.

## Abbreviations

FO: Forward osmosis
CTA: Cellulose triacetate
CDA: Cellulose diacetate
CNTs: Modified carbon nanotubes
SEM: Scanning electron microscopy
AFM: Atomic force microscopy
FT-IR: Fourier transform infrared spectroscopy
SW: Slaughter wastewater
LMH: Liter per square meter per hour
A: Membrane area (m^2^)
W_d,t_: Weight of draw solution at time t (g)
W_d,0_: Weight of draw solution at time zero (g)
W_f,0_: Weight of feeding solution at time zero (g)
J_w_: Water flux
SR: Salt rejection
C_p_: Permeate concentration
C_f_: Feed solution concentration
ρ_w_: Water density
ROI: Region of interest
ICP: Internal concentration polarization
S_q_: Root mean square roughness
S_a_: Arithmetic average roughness
S_t_: Total roughness
π: Osmotic pressure
i: Dissociation factor
C: Molar concentration of draw solution
R: Gas constant
T: Absolute temperature
